# The Complex Dynamics of Violence and Burnout in Healthcare: A Closer Look at Physicians and Nurses

**DOI:** 10.1111/inr.70075

**Published:** 2025-07-18

**Authors:** Orhan Çakır, İrfan Akkoç, Korhan Arun, Ebru Dığrak, Hasibe Serap Uluırmak Ünlüeroğlugil

**Affiliations:** ^1^ Izmir Tinaztepe University Izmir Türkiye; ^2^ Tekirdag Namik Kemal University Tekirdag Türkiye; ^3^ Department of Nursing Faculty of Health Sciences, Izmir University of Economics Izmir Türkiye

**Keywords:** Burnout, fear of future violence, nurse, physician, violent events at work

## Abstract

**Aims and objectives:**

This study aims to examine the mediating role of fear of future violence in the relationship between exposure to violent events at work and burnout among physicians and nurses.

**Background:**

Workplace violence is an escalating concern within healthcare institutions in Türkiye. Increasing exposure to violent events among healthcare professionals contributes to heightened fear of future violence, which may in turn exacerbate burnout.

**Methods:**

A cross‐sectional research design was utilized, collecting data via an online survey distributed to 387 physicians and nurses. The hypothesized model was analyzed using structural equation modeling techniques implemented in AMOS and SmartPLS software.

**Results:**

The analysis revealed that exposure to workplace violence significantly elevated the depersonalization and emotional exhaustion dimensions of burnout. Conversely, it was associated with a paradoxical decrease in the personal accomplishment dimension. Notably, no statistically significant relationship was found between exposure to violence and fear of future violence. These findings underscore the complex interplay between various burnout dimensions and the impact of workplace violence on physicians and nurses.

**Discussion and conclusion:**

Workplace violence emerges as a significant contributor to burnout among physicians and nurses, affecting emotional exhaustion, depersonalization, and personal accomplishment in multifaceted ways. However, the anticipated relationship between exposure to violence and fear of future violence was not supported by the data, indicating a need for further research in this area.

**Implications for nursing policy:**

The findings of this study indicate that health policies in Türkiye should prioritize strategies aimed at preventing and managing violence against physicians and nurses. Measures such as violence management training, strengthening organizational support systems, and implementing clear protocols for reporting and responding to violent incidents can help mitigate burnout among physicians and nurses.

## 1 Introduction

Researching workplace violence among healthcare professionals is vital because its effects extend beyond physical harm, influencing their emotional health, job satisfaction, and interactions with colleagues, ultimately impacting the entire healthcare system (Lanctôt and Guay 2014). Over 70% of nurses experience various forms of workplace violence from different sources monthly, with patients and their relatives being the most common perpetrators of violence (Wang et al. 2022). The same authors underscored the critical necessity of addressing workplace violence as a risk factor for burnout among healthcare workers, especially nurses. However, even if antecedents and consequences of workplace violence have been examined in detail, exposure to violence has not been studied to the same extent among nurses (Havaei 2021; Havaei and MacPhee 2021). Moreover, in the everyday life of healthcare professionals, victimization by violence, particularly by strangers (i.e., violence not resulting from acquiescence), can feel relatively distant to nurses and doctors (Chadee 2022). As such, the construal of stranger‐perpetrated violence is often abstract and situated at a high level of construal, with a possible focus on its causes rather than its consequences. Consequently, not only direct exposure to violence (e.g., proximate or imminent violent events) but also the fear of future violence (i.e., anticipated or distant threats) can be important for nurses in terms of possible consequences. Therefore, it is crucial for the literature to address not only the causes and lived experiences of workplace violence but also its consequences and anticipated effects.

Workplace violence, as defined by the Occupational Safety and Health Administration and the International Labor Organization, encompasses acts or threats of physical violence, harassment, intimidation, or disruptive behavior that occur in the work environment (Kim et al. 2018; Pien et al. 2015). Exposure to violence, on the other hand, can occur through direct firsthand involvement, indirect secondhand witnessing, or a combination of both (Havaei 2021). Exposure to violence in various contexts (i.e., ethnic‐political, school, and family settings) has been associated with aggression and post‐traumatic stress, even after controlling for a range of demographic characteristics (Docherty et al. 2023; Landau 2010).

Some occupational stressors may be inherent to the healthcare profession, such as a high workload and work tempo, unusually long work hours, shift work, and hazardous environmental or ergonomic conditions. Therefore, it is often difficult to isolate these stressors. In contrast, workplace violence is a direct interpersonal stressor that activates the body's physiological systems in preparation for a fight–flight response (Rogelberg 2007). Furthermore, Lim et al. (2024) discussed the complex interplay between burnout, workplace violence, moral injury, and workforce attrition among emergency physicians, emphasizing the urgent need for action to address these issues.

In the context of healthcare, construal level theory (CLT) can provide valuable insights into how healthcare professionals perceive and respond to various aspects of their work environment, patient care, and organizational dynamics. CLT can be applied to understand how healthcare professionals cope with workplace violence by considering the mental construal levels of nurses and doctors as they process and interpret workplace violence (Kim and John 2008). The specific relevance of this theory will be elaborated on in the hypotheses section.

The impact of burnout on healthcare outcomes is substantial and far‐reaching, affecting patient care, nurse well‐being, and organizational dynamics. Research consistently demonstrates that nurse burnout is strongly associated with adverse patient outcomes, including negative patient experiences, decreased quality of care, and compromised patient safety (Nantsupawat et al. 2016; Poghosyan et al. 2010). Additionally, nurse burnout is linked to low staffing levels and unfavorable practice environments, which further diminish the quality of patient care (Poghosyan et al. 2010). Since nursing staff are often the primary healthcare professionals with whom patients interact during treatment, the prevalence of burnout among nurses directly impacts the quality of care delivered (Rezaei et al. 2018).

Furthermore, burnout has a substantial impact on nurse performance outcomes, with studies indicating that it is associated with reduced nursing performance and professionalism (An et al. 2020). This may lead to decreased quality of nursing care, compromised patient outcomes, and increased turnover rates among nursing staff (Luan et al. 2017). The repercussions of burnout extend beyond individual nurses, affecting the overall functioning of healthcare systems and highlighting the critical need to address and mitigate burnout among nursing professionals (Qedair et al. 2022).

In a nutshell, this study investigates the effects of workplace violence on nurses’ and physicians’ burnout, with the mediating role of the fear of future violence. It makes four major contributions to literature and practice. First, the consequences of workplace violence and exposure to violence can manifest in various ways, affecting individuals’ emotional well‐being, stress levels, and burnout (Mayhew and Chappell 2007; Shahrour et al. 2022; Teymourzadeh et al. 2014). Therefore, it is essential to examine the effects of exposure to violence on burnout, rather than solely on workplace violence. Second, to the authors’ knowledge, this is the first study to explore the relationship between fear of future violence and exposure to violence among nurses. Third, the context of violence has been examined through the lens of CLT. Finally, fear of future violence is introduced for the first time as a mediating variable between exposure to violence and burnout.

The objective of this study is to examine the mediating role of fear of future violence in the relationship between exposure to violent incidents at work and burnout (measured across three dimensions: emotional exhaustion, depersonalization, and personal accomplishment) among healthcare professionals, particularly physicians and nurses. This research employs a cross‐sectional design and utilizes structural equation modeling to analyze data collected from 387 participants.

## 2 Background

2.1

CLT is a psychological theory that explains how psychological distance, the perceived distance between an individual and an event, which can be temporal, spatial, social, or hypothetical, affects the way the event is mentally represented (Yan et al. 2014). More specifically, CLT provides a framework for understanding how perceived psychological distance influences mental representations of events: distant events are construed more abstractly, while near events are construed more concretely (Medvedev 2022; Ram et al. 2024). According to CLT, events perceived as distant are represented in a more abstract manner, focusing on essential, high‐level features, whereas events perceived as near are represented in a more concrete manner, emphasizing specific, low‐level details (Stillman et al. 2020; Wang et al. 2021). This theory helps explain how people think about and make decisions regarding events that vary in temporal, spatial, social, or hypothetical distance (Brügger 2020).

More clearly, CLT suggests that psychological distance influences how individuals perceive and interpret events. It suggests that events perceived as distant are conceptualized in abstract terms, while those perceived as closer are thought of in more concrete terms. This is particularly relevant in high‐stress environments like healthcare settings, where fear of future violence can shape emotional responses and coping mechanisms. Abstract thinking emphasizes systemic issues contributing to workplace violence, while concrete thinking emphasizes individual experiences, potentially leading to avoidance behaviors. As anticipated, violence recedes, and healthcare professionals may employ more abstract thinking, leading to resilience‐building. However, if expectations of future violence persist, it can exacerbate mental health issues and emotional exhaustion. Temporal distance refers to future impacts or thinking about the future. Healthcare professionals may adopt abstract thinking and develop coping strategies as anticipated violence recedes, leading to resilience‐building. However, if violence remains imminent, it can worsen mental health issues, including burnout, anxiety, and depression among healthcare professionals. Healthcare professionals’ perception of workplace violence impacts their emotional responses and coping mechanisms. Frequent violence may cause anxiety and fear, while rare or distant violence may lead to abstract safety protocols or workplace improvements. Abstract thinking focuses on systemic workplace violence, promoting long‐term strategies like crisis deescalation training, while concrete thinking emphasizes individual experiences, potentially causing fear and anxiety, affecting healthcare workers’ well‐being and performance (Holmes et al. 2011;  Trope and Liberman 2011).

This means that when healthcare professionals perceive violence as psychologically distant, they may think about it in a more abstract and generalized manner, potentially downplaying its immediacy and impact on their emotional state. Conversely, when violence is perceived as close or imminent, it is processed in a more detailed and specific way, which can heighten emotional responses and perceptions of threat (Slepian et al. 2015). As temporal distance increases, events are represented more abstractly, focusing on their essential features rather than concrete details. Events perceived as spatially distant are construed at a higher level, emphasizing their central and abstract characteristics (Fiedler et al. 2012). This can lead to a more detached perception of violence occurring far away, potentially influencing judgments and behaviors related to those events. Thus, CLT provides a framework for understanding how varying levels of psychological distance can shape the emotional and cognitive responses of healthcare workers to violence in their environments.

### 2.2 Effects of Exposure to Violence on Burnout

Studies across the literature have highlighted the profound impact of workplace violence on burnout among healthcare professionals. For instance, Roldán et al. (2013) and Jeon et al. (2017) identified significant associations between workplace violence and burnout levels among healthcare workers, negatively affecting job satisfaction and mental health. Moreover, research by Wang et al. (2024) and Lim et al. (2024) emphasizes the mediating roles of stress and insomnia in the relationship between workplace violence and burnout, underscoring the urgent need for intervention strategies to mitigate these effects. Anbesaw et al. (2023) investigated burnout syndrome among healthcare professionals in Ethiopia, revealing a significant association between experiences of physical violence and burnout. Duan et al. (2019) studied the impact of workplace violence on job satisfaction, burnout, and turnover intention among healthcare workers, highlighting the correlation between emotional exhaustion and various forms of violence. Roldán et al. (2013) found that a high frequency of violence is associated with increased levels of burnout, identifying violence as a significant risk factor that substantially raises the likelihood of burnout among those who experience regular assaults.

A recent study highlighted the direct link between violence against nurses in healthcare settings and burnout, underscoring its detrimental effects on individual safety, well‐being, and job performance (Molero Jurado et al. 2023). The findings of Fei et al. (2023) indicate that workplace violence is significantly associated with higher levels of burnout and its components. The authors reported a significant positive correlation between workplace violence and overall burnout (β = 0.273, *p* < 0.001), suggesting that increased exposure to workplace violence is linked to more severe burnout symptoms. Furthermore, workplace violence was positively correlated with emotional exhaustion (β = 0.285) and depersonalization (β = 0.293), indicating that experiences of violence contribute specifically to these dimensions of burnout. High and medium frequencies of violence were also associated with higher levels of burnout, with assaults increasing the risk 1.4 and 1.9 times, respectively, compared with individuals who had not been assaulted (Estryn‐Behar et al. 2008). Research by Roldán et al. (2013) also demonstrated a positive relationship between violence and burnout, with aggression causing high levels of anxiety and depression among nurses, with 50.0% reported anxiety and 37.5% reported depression. These findings are especially important, as some studies suggest a strong correlation between burnout, depression, and anxiety, indicating that burnout may, in some cases, be a manifestation of depression and anxiety (Koutsimani et al. 2019; Ernst et al. 2021).

Burnout is rooted in cognitive and emotional factors such as distress and maladaptive schemas (Bay and Novin Rouz 2022). Additionally, the cognitive and emotional impacts of workplace violence on nurses are substantial. Workplace violence triggers cognitive responses, prompting nurses to develop coping mechanisms. However, these mechanisms do not necessarily prevent increased levels of burnout (Boyle and McKenna 2016; Erkutlu et al. 2024). Moreover, Montgomery et al. (2021) found that nurses who reported high levels of burnout were particularly affected by emotional exhaustion.

An investigation conducted in Palestinian hospitals found a significant correlation between exposure to physical violence and a heightened level of burnout among emergency department personnel (Hamdan and Hamra 2017). Furthermore, research by Pai et al. (2015) revealed a link between the experiences of both physical and verbal violence and the development of burnout symptoms and mild psychiatric issues among healthcare professionals. Additionally, when nurses are the direct victims, indirect victims, or witnesses of the violence, such experiences are considered forms of exposure to workplace violence. These results can be categorized under the same conceptual framework (Byon et al. 2022). More specifically, workplace violence, whether direct or indirect and targeting nurses, can be studied as exposure to violence.

**H1**: Exposure to violence increases burnout among physicians and nurses.

### 2.3 The Mediating Role of Fear of Future Violence in the Relationship Between Exposure to Violence and Burnout

To examine the mediating effect of the fear of future violence, it is necessary to analyze the direct relationship between exposure to violence and fear of future violence, as well as the direct relationship between fear of future violence and burnout. Exposure to violence can lead to a range of psychological and physical consequences, including fear (Atawneh et al. 2003; Kafle et al. 2022). This fear may take various forms, including fear of future violence (Rogers and Kelloway 1997). Specifically, Gedik et al. (2023) suggest that the frequency of violence can increase fear of future violence among healthcare employees. While some studies have explored the relationship between nurses’ resilience and exposure to violence, their findings are inconsistent, highlighting the need for further research in this area (Sani et al. 2020). Research by Xu et al. (2022) emphasizes that psychological resilience plays a protective role in coping with workplace violence and its psychological effects. Therefore, it can be argued that more resilient nurses are less vulnerable to developing fear of future violence, a topic that warrants further investigation.

The relationship between CLT and fear of future violence can be understood through the lens of psychological distance and mental representation. According to CLT, individuals’ mental construal of information, events, and actions varies depending on perceived psychological distance. When events are perceived as distant, they are mentally represented at a higher construal level, focusing on abstract and generalized features. In the context of fear of future violence, individuals may construe potential threats at a higher level, emphasizing abstract notions of danger and its broader implications. This elevated construal level can contribute to increased fear and anxiety about potential violent situations, as individuals focus more on the generalized consequences and less on specific, immediate details.

On the other hand, CLT suggests that how nurses perceive and interpret the threat of violence may depend on the psychological distance of the perceived risk. In other words, if they are not directly involved in violent incidents or exposed to nearby violence, they may perceive such threats as more abstract rather than immediate or realistic. For example, Santos and Beuren (2022) found that coercive practices, as opposed to enabling management systems, are represented more concretely, that is, lower level of construal. This suggests that nurses may tend to downplay or forget negative or coercive behaviors from patients and instead focus on acts of kindness. As a result, this selective perception may reduce their fear of future violence.

However, in the Turkish context, where violence is more prevalent, it is believed that violence against nurses is more severe, leading to heightened levels of fears (Aytac et al. 2011; Prajapati et al. 2023). Results from Akbolat et al. (2021) conducted in Türkiye support this perspective.

**H2**: Exposure to violence increases fear of future violence among physicians and nurses.

### 2.4 The Effects of Fear of Future Violence on Burnout Among Nurses

Workplace violence and burnout among healthcare personnel have been extensively studied (Jeon et al. 2017). Workplace violence can lead to exhaustion, job dissatisfaction, and mental health problems such as depression and post‐traumatic stress disorder (Homotoff 2021). Yoon and Sok (2016) investigated violence, burnout, and job satisfaction among Korean nurses in emergency care centers, finding that exposure to violence had a substantial impact on burnout. Moreover, exposure to workplace violence has been shown to predict anxiety about future violence, which in turn predicts psychological well‐being and somatic symptoms (Rogers and Kelloway 1997). Nurses who fear workplace violence are more likely to experience emotional exhaustion, cynicism, and a reduced sense of personal accomplishment. Thus, similar to actual workplace violence, fear of future violence can also be a significant predictor of fatigue. A recent study indicated that fear of future workplace violence is strongly associated with nurse burnout (Fu et al. 2021). Similarly, Akbolat et al. (2021) found that both direct and witnessed workplace violence significantly impact healthcare workers’ fear of future violent incidents and turnover intention.

**H3**: Fear of future violence increases burnout among physicians and nurses.

Similarly, Jeon et al. (2017) revealed a positive correlation between overall workplace violence, including verbal abuse and physical threats from patients and caregivers with and burnout among dental hygienists. Wang et al. (2024) found that workplace violence significantly predicted job burnout, with stress and insomnia acting as mediators in this relationship. Gérain and Zech (2022) identified exposure to workplace violence as an environmental factor contributing to burnout risk. Likewise, Thompson et al. (2023) emphasized the importance of addressing workplace violence in order to mitigate its impact on healthcare professionals’ well‐being and job satisfaction.

## 3 Methods

### 3.1 Design

A cross‐sectional study investigated the mediating role of fear of future violence in the relationship between workplace violence and burnout among physicians and nurses.

### 3.2 Sampling and Participants

The survey was administered online via Google Forms, targeting physicians and nurses in İzmir, Türkiye. Participants were presented with an informed consent statement prior to beginning the survey. The total population of potential participants was approximately 25,000 healthcare professionals. Based on the guidelines provided by Sekaran and Bougie (2016), a sample size of 378 was determined to be adequate for normal distribution, using a 5% margin of error and a 95% confidence range. Participants were selected using a convenience sampling method. Data were collected from 387 participants, including 176 physicians and 211 nurses. Analyses were conducted using this dataset. Demographic data revealed that 60.5% of participants were female (*n* = 234), 45.5% were physicians (*n* = 176), 52.2% were unmarried (*n* = 202), 44.2% held a PhD (*n* = 171), and 84.2% were employed in the public sector (*n* = 326). The mean age of participants was 32.49 years (SD = 7.67), and the average work experience was 9.19 years (SD = 7.86).

### 3.3 Instruments

Data were collected using the Personal Information Form, the Fear of Future Violence Scale, the Exposure to Violence Scale, and the Burnout Scale.

The Personal Information Form, developed by the researchers, consists of seven sociodemographic questions related to age, gender, marital status, educational status, years of work experience, department, shift work status, and numbers of shifts.

The Exposure to Violence Scale was developed by Rogers and Kelloway (1997) to assess workplace violence. It comprises two dimensions: direct violence at work (8 items) and witnessed violence at work (5 items). Akbolat et al. (2021) validated the Turkish version of the scale, reporting reliability coefficients of 0.72 and 0.91, respectively. Items are rated on a 5‐point Likert scale (1 = never, 5 = four or more times).

The Fear of Future Violence Scale was developed by Rogers and Kelloway (1997) and measures employees’ fear of experiencing violence at work in the future. The scale includes 10 items that address the potential for both physical and nonphysical violence within the coming year. Higher mean scores indicate greater fear of anticipated violence. Akbolat et al. (2021) reported a reliability coefficient of 0.94 for the Turkish version. The scale uses a 5‐point Likert scale (1 = strongly disagree, 5 = strongly agree).

The Maslach Burnout Inventory was developed by Maslach and Jackson (1981). The Turkish validity and reliability study of the scale was conducted by Ergin (1992). The scale measures burnout using 22 items across three dimensions: emotional exhaustion (9 items), depersonalization (5 items), and personal accomplishment (8 items). Responses are rated on a 5‐point Likert scale (1 = strongly disagree, 5 = strongly agree). The Cronbach's alpha coefficients for the Turkish version were reported as 0.83 for emotional exhaustion, 0.65 for depersonalization, and 0.72 for personal accomplishment.

### 3.4 Data Collection

This part of the survey data was collected through a self‐report questionnaire conducted in İzmir, Türkiye, between September 2023 and July 2024. The participants were registered physicians and nurses with experience in the healthcare sector. A convenience sampling strategy was used to distribute the survey to physicians and nurses, resulting in 387 valid responses selected for data analysis. The survey link was disseminated by the authors via social media platforms, professional networks, institutional email lists, and direct invitations to potential participants. Prior to beginning the survey, participants were required to read an informed consent statement presented at the start of the online questionnaire. This statement provided information about the purpose of the study, the voluntary nature of participation, the confidentiality of responses, and the intended use of the collected data. Only those who provided informed consent through the form on Google Forms were allowed to proceed with the survey.

### 3.5 Statistical Analysis

Data analysis was conducted using the Statistical Package for the Social Sciences (SPSS), version 21, with a 95% confidence interval applied to determine statistical significance. Confirmatory factor analysis (CFA) was performed to assess the structural validity of the study model, employing the maximum likelihood estimation method. The Cronbach's alpha reliability coefficient was calculated to evaluate the internal consistency of the scales. Descriptive statistics were used to analyze the sociodemographic data. Pearson correlation analysis was conducted to examine the relationship among the study variables. To test the mediation model, preliminary analyses were followed by structural equation modeling (SEM) using both AMOS and SmartPLS. The mediation model was validated through these SEM analyses.

### 3.6 Ethical Considerations

This study was approved by the Health Sciences Research Ethics Committee of the relevant university (Approval Date: March 3, 2023; Decision No: 05.05‐20‐213). Participants were informed through a written consent form presented at the beginning of the survey, prior to their participation in the study. In addition, authorization was obtained from the institutions where the research was conducted.

## 4 Results

### 4.1 Common Method Bias

The use of a single‐source survey method in this study raises potential concerns regarding common method bias (CMB). To address this issue, Harman's single‐factor test was conducted by examining the first eigenvalue derived from a factor analysis using SPSS. CMB is considered a concern if the first unrotated factor accounts for a substantial portion of the total variance. In this analysis, the first factor accounted for only 27.67% of the total variance, which is well below the 50% threshold recommended by Fuller et al. (2016). Therefore, it is unlikely that CMB significantly influenced the results of this study.

### 4.2 Measurement Validation

The measurement model in this study includes three variables: violent events at work (13 items), fear of future violence (10 items), and burnout (22 items). Results from the CFA indicate that all factor loadings are statistically significant, ranging from 0.52 to 0.98. The goodness‐of‐fit indices demonstrate an acceptable fit between the model and the data: χ^2^/df = 3.01, *p* < 0.001, CFI = 0.91, TLI = 0.91, RMSEA = 0.07, and SRMR = 0.08 (Hu and Bentler 1999; Hair et al. 2016).

All standardized factor loadings were statistically significant, with each exceeding0.50. However, three items within the burnout variable exhibited loading estimates below 0.50, which negatively impacted the construct's average variance extracted (AVE), keeping it below the recommended 0.50 level. These items were removed to improve the model fit and ensure validity. Following their removal, CFA results showed that the AVE for each construct exceeded the suggested threshold of 0.50, thereby confirming convergent validity (Hair et al. 2016).

Furthermore, composite reliability values for each construct exceeded the recommended threshold of 0.70, providing additional support for convergent validity (Fornell and Larcker 1981). To assess discriminant validity, the AVE criterion was applied. As presented in Table 1, the AVE values for each variable are higher than the squared correlations between variables, indicating that discriminant validity is established within the model.

**TABLE 1:** Reliability and correlation values (*N* = 387).

Variables	CA	CR	AVE	*M*	SD	1	2	3	4	5
1. Direct violence at work	0.91	0.93	0.62	1.73	0.89					
2. Witnessed violence at work	0.87	0.90	0.65	3.29	1.26	0.57^*^				
3. Emotional exhaustion	0.94	0.95	0.70	2.67	1.25	0.38^**^	0.43^**^			
4. Personal accomplishment	0.80	0.86	0.56	4.22	0.76	−0.21^**^	−0.08	−0.34^**^		
5. Depersonalization	0.89	0.92	0.58	2.52	0.99	0.39^**^	0.35^**^	0.70^**^	−0.24^**^	
6. Fear of future violence	0.99	0.99	0.89	4.25	1.09	−0.03	0.21^**^	−0.07	0.52^**^	0.01

*Notes*: CA = Cronbach's alpha; CR = composite reliability; AVE = average variance extracted; M = mean; SD = standard deviation

^*^ *p* < 0.05 (two‐tailed)

^**^ *p* < 0.01 (two‐tailed).

### 4.3 Reliability and Correlation Analysis

Table 1 presents the reliability coefficients, means, standard deviations, and correlation analysis results for data collected from physicians and nurses regarding the variables of violent events at work, fear of future violence, and burnout. The research model revealed significant relationships among the dependent, independent, and mediator variables. Based on the correlation values in Table 1, significant positive correlations were found between direct workplace violence and emotional exhaustion (*r* = 0.38, *p* < 0.01), as well as between witnessed workplace violence and emotional exhaustion (*r* = 0.43, *p* < 0.01). Conversely, a significant negative correlation exists between direct workplace violence and personal accomplishment (*r* = −0.21, *p* < 0.01), whereas no significant relationship was observed between witnessed workplace violence and personal accomplishment (*r* = −0.08, *p* = 0.12). Depersonalization showed significant positive correlations with both direct workplace violence (*r* = 0.39, *p* < 0.01) and witnessed workplace violence (*r* = 0.35, *p* < 0.01). The Fear of Future Violence Scale demonstrated significant positive correlations with witnessed workplace violence (*r* = 0.21, *p* < 0.01) and personal accomplishment (*r* = 0.52, *p* < 0.01), but no significant correlations with other variables. These findings suggest that perceptions of violent events at work are related to fear of future violence and burnout among healthcare professionals.

Succinctly, the correlations table (Table 1) shows that emotional exhaustion and depersonalization are closely linked, both being significantly influenced by exposure to violence. Personal accomplishment appears to serve as a protective factor against burnout, while fear of future violence does not exhibit strong correlations with the other constructs. In summary, the data highlight the significant relationships between emotional exhaustion, depersonalization, and exposure to violence, whereas personal accomplishment emerges as a potential buffer against burnout.

### 4.4 Test of Hypotheses

**FIGURE 1:**
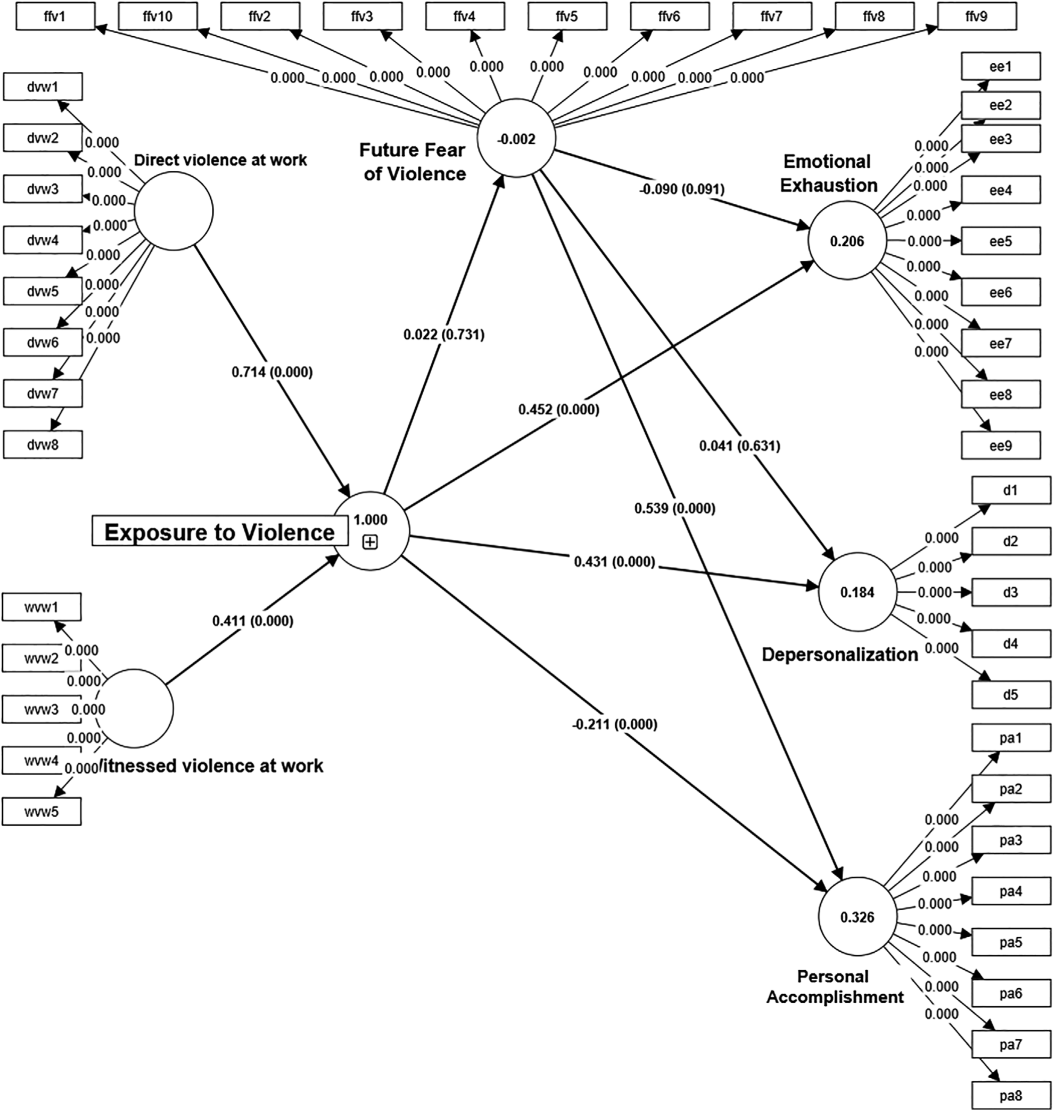
Structural equation model findings.

The SEM analysis conducted using SmartPLS revealed significant findings regarding the impact of exposure to violence on various dimensions of burnout among physicians and nurses (Figure 1). The results indicated that exposure to violence significantly increases both depersonalization (β = 0.43, *p* < 0.05) and emotional exhaustion (β = 0.45, *p* < 0.05), which are core dimensions of burnout. Interestingly, exposure to violence was also found to decrease the reduced personal accomplishment dimension of burnout (β = −0.22, *p* < 0.05), suggesting a potential counterintuitive effect. These findings indicate that Hypothesis 1 is partially supported. Despite the theoretical expectation of a direct link between exposure to violence and fear of future violence, the analysis revealed that this relationship is not statistically significant (β = 0.80, *p* > 0.05). Therefore, Hypothesis 2 is not supported. The study further explored the impact of fear of future violence on the three burnout dimensions. Results showed that fear of future violence does not significantly affect emotional exhaustion and depersonalization (*p* > 0.05). However, it has a significant positive effect on the reduced personal accomplishment dimension (β = 0.54, *p* < 0.05). Accordingly, Hypothesis 3 is not supported.

This study investigates whether physicians’ and nurses’ perceptions of direct violence at work, witnessed violence at work, fear of future violence, emotional exhaustion, depersonalization, and personal accomplishment differ significantly based on their professional title. An independent samples *t* test was conducted on data from 387 participants (176 physicians and 211 nurses). The results revealed substantial differences between the two professional groups across all examined variables. Nurses reported significantly higher mean scores for direct violence at work (*t* = −4.43; *p* < 0.001, M = 1.91; SD = 1.05) and witnessed violence at work (*t* = −2.58; *p* < 0.05, M = 3.44; SD = 1.29) than physicians (M = 1.52; SD = 0.60 and M = 3.11; SD = 1.19, respectively). Nurses also reported significantly higher levels of emotional exhaustion (*t* = −9.99; *p* < 0.001, M = 3.19; SD = 1.14) and depersonalization (*t* = −6.60; *p* < 0.001, M = 2.81; SD = 1.03) compared with physicians (M = 2.05; SD = 1.08 and M = 2.17; SD = 0.85, respectively). Conversely, physicians reported significantly higher levels of fear of future violence (*t* = 6.32; *p* < 0.001, M = 4.62; SD = 0.81) and personal accomplishment (*t* = 7.48; *p* < 0.001, M = 4.51; SD = 0.59) than nurses (M = 3.94; SD = 1.21 and M = 3.97; SD = 0.80, respectively). These findings indicate that, while nurses experience more frequent and intense negative workplace experiences, such as exposure to violence, emotional exhaustion, and depersonalization, physicians exhibit a higher fear of future violence but also report a stronger sense of personal accomplishment in their professional roles.

## 5 Discussion

According to the SEM analysis conducted using SmartPLS, exposure to violence significantly increases both the depersonalization (β = 0.43, *p* < 0.05) and emotional exhaustion (β = 0.45, *p* < 0.05) dimensions of burnout. However, interestingly, it increases personal accomplishment dimension (β = −0.22, *p* < 0.05). Although these dimensions are commonly assessed together to provide a comprehensive measure of burnout, the findings suggest that the interplay between emotional exhaustion, depersonalization, and reduced personal accomplishment may not fully capture the burnout experience among healthcare professionals in Türkiye.

The impact of workplace violence on healthcare professionals is substantial. Various studies have demonstrated that such workplace violence contributes to burnout, particularly by reducing personal accomplishment (Ahmed Higazee and Rayan 2017; Liu et al. 2018). For example, research on emergency department personnel revealed that individuals experiencing burnout, particularly in terms of emotional exhaustion and depersonalization, were more likely to report higher turnover intentions, a factor closely associated with reduced personal accomplishment (Hamdan and Hamra 2017). Additionally, healthcare professionals who had been exposed to workplace violence and who reported high levels of fear of future violence experienced lower levels of personal accomplishment compared with those who reported less fear (Molero Jurado et al. 2023). Moreover, nursing interns subjected to workplace violence also reported diminished personal accomplishment (Yang et al. 2023).

These findings underscore the adverse effects of workplace violence on healthcare professionals’ sense of personal achievement and fulfillment in their roles. However, it is essential to consider contextual and individual factors when examining the relationship between workplace violence and motivation, as organizational support and safe work environments can play a crucial role in mitigating the negative effects of violence (Cakal et al. 2021; Yoon and Jung‐Choi 2021). For example, interventions such as simulation training aimed at managing workplace violence have been shown to increase confidence and enhance nursing staff skills, which may, in turn, boost motivation (Ming et al. 2019). Emmerling (2024) emphasized the importance of implementing programs to prevent workplace violence, supporting the view that training is essential. These programs help nurses and physicians manage patient aggression and improve overall workplace safety. By implementing such initiatives, healthcare professionals can gain greater confidence in handling violent situations. Many studies have described the high prevalence of verbal and physical violence experienced by nurses and doctors in Türkiye, but they often fail to offer tailored, preventive strategies (Hamzaoglu and Türk 2019; Pinar and Ucmak 2011; Şenuzun Ergün and Karadakovan 2005; Atan et al. 2013). Therefore, this study offers a new perspective for Turkish healthcare professionals, highlighting that training plays a critical role, even though it has often occurred without clear strategic planning or direction from individual hospital management teams.

Another interesting finding is that exposure to violence was not statistically significant in predicting fear of future violence among Turkish healthcare professionals (β = 0.80, *p* > 0.05). Although prior literature has suggested a correlation between exposure to violence and the development of fear of future violence, particularly in healthcare settings, our results did not support this relationship. Research on the effects of exposure to violence on fear and aggression has produced mixed results. Some studies indicate that workplace violence can increase fear of future violence and lead to various negative psychological outcomes (Rogers and Kelloway 1997). However, other research has found that exposure to community violence does not significantly influence emotional distress or future violent behavior, particularly among boys (Farrell and Bruce 1997). In Türkiye, workplace violence against nurses and doctors remains a significant problem, with reported prevalence rates ranging from 44.7% to 64.1% (Pinar et al. 2017). However,  workplace violence exposure rates may be even higher, ranging from 24.6% to 85% (Sahi̇p et al. 2021). Various factors have been identified influencing fear of future violence, including gender, marital status, income, department, and previous experience with violence (Fu et al. 2021). Cultural influences significantly influence workplace violence perception and response in developing countries like Türkiye. Normalization of violence can lead to desensitization, reducing perceived burnout or motivation levels, despite the expected negative correlation with violence. For example, Salvador et al. (2021) found that cultural stigma can lead to underreporting and inadequate institutional responses, perpetuating the cycle of violence and psychological distress. Different perceptions of patient interaction can also vary based on cultural backgrounds, causing frustrations and potentially leading to aggression. Moreover, in some developing countries, both healthcare personnel and patients frequently show a lack of cultural respect for the nursing profession, which can create conditions conducive to violence (Dafny et al. 2024).

Additionally, ruminative thinking following such exposure may exacerbate the negative psychological effects (Niven et al. 2013), underscoring the importance of addressing cognitive and emotional responses to workplace violence (Fu et al. 2021). The CLT may help to understand the reciprocal relationship between psychological distance and mental representation, emphasizing that healthcare professionals’ perceptions and responses to workplace violence are influenced by their construal levels, which can lead to a focus on immediate safety rather than systemic solutions, particularly in the context of cultural attitudes prevalent in Türkiye.

The CLT proposes a reciprocal relationship between psychological distance and levels of mental representation (Henderson and Wakslak 2010). The theory has been applied across various disciplines, including criminology, where it offers insights into the fear of crime and perceptions of criminal events (Gouseti 2015). CLT is relevant to numerous psychological processes, such as anticipation, evaluation, and self‐regulation (Lee 2019). Henderson and Wakslak (2010) explored CLT's application to physical distance, examining the factors that influence distance perception and how distance affects mental representation, judgment, and behavior. In the context of workplace violence, individuals operating at a high level of construal may interpret violent incidents in more abstract terms, considering their broader implications for the work environment, organizational culture, and professional relationships. The attitudes and responses of healthcare professionals toward workplace violence may be influenced by factors such as professional training, organizational policies, and personal experiences with violence (Dafny et al. 2023). Additionally, healthcare professionals often reflect the broader cultural values and societal attitudes toward abuse that are prevalent in their communities (Gümüşsoy et al. 2021; Zorjan et al. 2017). In Türkiye, for example, some nurses may hesitate to intervene in violent incidents involving colleagues, influenced by their personal attitudes toward domestic violence (Burçin Biçici and Toraman 2014). However, these attitudes do not always translate into concrete action when violence occurs. According to CLT, healthcare professionals may mentally represent such incidents at varying levels of construal, ranging from high‐level, abstract interpretations to low‐level, detail‐oriented representations.

These differing mental frameworks can influence how they perceive, emotionally respond to and choose to act in situations involving workplace violence. Healthcare professionals may interpret and respond to such incidents of workplace violence based on their construal levels. For example, individuals operating at a high construal level may focus on the broader implications of violence, such as its impact on the work environment and patient care. In contrast, those with a low construal level are more likely to concentrate on the immediate, concrete details of violent encounters. Consequently, we can say that fear of future violence does not affect burnout due to the low construal level, related to the cultural settings of healthcare professionals. As a result, in Türkiye, healthcare professionals prioritize immediate responses and personal safety measures rather than seeking systemic solutions and organizational support according to CLT (Zhao et al. 2015).

Workplace violence is widely recognized as a significant occupational hazard, especially in developing countries, where it disproportionately affects healthcare professionals (Ahmed et al. 2022). Compared with developed countries, developing countries generally lack comprehensive and mandatory legislation to assess and prevent psychosocial risks and workplace violence (Chirico et al. 2019). However, our results show that Türkiye stands out among developing countries by having more concrete and updated mandatory legislation aimed at preventing workplace violence. Despite these legal frameworks, a contrasting reality emerges: many healthcare professionals in Türkiye report feeling unsupported in their work environments and frequently experience occupational burnout (Çevik et al. 2020). This discrepancy points to a gap between policy and practice, indicating that legislation alone may be insufficient without effective implementation, organizational support, and a supportive workplace culture.

Fear of future violence has little effect on two aspects of burnout: emotional exhaustion and depersonalization (*p* > 0.05). Fear of future violence significantly reduces personal accomplishment (*p* < 0.05, β = 0.54). As a result, H3 is rejected because fear of future violence only affects one dimension of burnout. Workplace violence is a critical issue that may be shaped by cultural dynamics (Dillon 2012). In particular, cultural factors such as patriarchal norms and low educational levels have been linked to the persistence of violence (Ökten 2017). Historical events and political instability have also contributed to a broader culture of violence within Turkish society (Gawrych 1986). Moreover, fear and personal values play crucial roles in shaping both individual and collective responses to violence (Eren 2005). Emotional exhaustion refers to feelings of being emotionally overextended and physically depleted, while depersonalization involves developing cynicism and emotional detachment from one's work (Apikoglu and Hosgorur 2013).

Although previous studies suggest that emotional intelligence and job content significantly influence personal accomplishment (Cao et al. 2022; De Looff et al. 2018), this relationship may manifest differently among Turkish healthcare professionals. Typically, moderate levels of emotional exhaustion and depersonalization are linked to reduced feelings of personal accomplishment, especially among nurses involved in direct patient care, who are particularly susceptible to these burnout dimensions (Gilavandi et al. 2019). However, in the context of widespread workplace violence—an increasingly alarming issue in the Turkish healthcare system—nurses and physicians may not experience emotional exhaustion or depersonalization in conventional ways (Hamzaoglu and Türk 2019). Instead, their professional identity may remain intact or even become more resilient, shaped by a commitment to patient care in the face of adversity. Nevertheless, upholding high standards of competence, moral responsibility, compassion, integrity, and ethical practice remains critical to preserving both the professional identity of healthcare workers and the public's trust in the healthcare system (Liang et al. 2020).

Overall, the findings of this research indicate that workplace violence significantly contributes to burnout among healthcare professionals. The results of this research show that various dimensions of burnout and violence are multifaceted.

The findings of the *t*‐test indicate that nurses experience more frequent and intense negative workplace events, including direct violence, emotional exhaustion, and depersonalization. In contrast, physicians not only report higher levels of fear concerning future violence but also demonstrate a greater sense of personal accomplishment in their professional roles. This contrast underscores the distinct challenges and emotional dynamics faced by nurses and physicians, reflecting the varied occupational stressors within healthcare settings.

Nurses often experience more direct violence at work than physicians due to the nature of their roles and the environments in which they work. As frontline healthcare providers, nurses frequently engage with patients during moments of high stress, emotional instability, or medical crisis, conditions that can increase the risk of verbal and physical abuse (Cheung et al. 2018). In the current study, nurses reported significantly higher mean scores for witnessing violence at work compared with physicians (*t* = −2.58, *p* < 0.05), indicating a meaningful difference in the prevalence of such incidents between the two professional groups. This disparity may also be influenced by the tendency for underreporting among nurses, which is often attributed to complex judicial procedures and the lack of robust, supportive reporting mechanisms (Kaya et al. 2016). As a result, the actual incidence of violence witnessed by nurses may be even higher than reported figures suggest. From this perspective, our research sheds light on an important aspect of perceiving lower levels of organizational justice and support.

The results show that nurses reported significantly higher levels of emotional exhaustion compared with physicians (*t* = −9.99, *p* < 0.001). This indicates that nurses experience higher levels of emotional exhaustion, which may be linked to their exposure to violence and demanding work conditions. This result is supported by Renzi et al.’s (2012) work, which found that nurses experience higher levels of emotional exhaustion due to perceived insufficient recognition of personal commitments, limited managerial ability, and unsatisfactory communication.

Nurses also exhibited higher levels of depersonalization (M = 2.81, SD = 1.02) compared with physicians (M = 2.17, SD = 0.85), with a *t*‐statistic of −6.60 (*p* < 0.001). This suggests that nurses may develop a more detached or cynical attitude toward their patients, potentially as a coping mechanism in response to their stressful work environment.

In contrast, physicians reported a significantly higher mean score for fear of future violence (M = 4.62, SD = 0.81) compared with nurses (M = 3.95, SD = 1.21), with a *t*‐statistic of 6.32 (*p* < 0.001). This indicates that while nurses experience more direct violence, physicians are more concerned about the potential for future violent incidents. There is a general concern among healthcare workers about the potential for future violence, but specific comparative data on whether physicians are more concerned than nurses are not provided (Berlanda et al. 2019; Schiltz et al. 2020). Therefore, the results of this paper contribute significantly to the literature by providing the first comparison between nurses’ and physicians’ fear of future violence. Both physicians and nurses often do not report violent incidents due to the belief that it is part of the job, previous experiences of inaction, and fear of consequences (Algwaiz and Alghanim 2012; Kitaneh and Hamdan 2012). These results support the view that physicians either do not report the violence or avoid engaging with the associated paperwork.

Future research should focus on several key areas to enhance understanding and improve conditions for nurses worldwide. Firstly, understanding mediating factors such as organizational support, coping mechanisms, and resilience between violence and burnout could lead to targeted interventions. Secondly, further research is needed to examine cultural and healthcare contexts to develop effective strategies for enhancing nurse well‐being. Thirdly, the prevalence and impact of workplace violence against nurses is a pressing issue that transcends national boundaries. While studies report alarming rates of violence against healthcare professionals in Türkiye, similar patterns may exist in other countries. Future research should aim to conduct comparative studies that assess the prevalence of workplace violence across various healthcare systems and its psychological and professional consequences for nurses. Such studies could provide valuable insights into the global nature of this issue and inform policy changes aimed at protecting healthcare workers. Lastly, emotional intelligence and job satisfaction may help mitigate burnout among nurses, but their relationship may vary across cultural contexts. Future research should explore how emotional intelligence interacts with job content and environmental factors, potentially improving nurse retention and the quality of patient care.

### 5.1 Limitations

This study has several limitations that should be considered when interpreting the results. First, the cross‐sectional design limits the ability to establish causal relationships between variables. Second, the sample consisted solely of healthcare professionals in Türkiye, which may limit the generalizability of the findings to other contexts or countries. Additionally, reliance on self‐reported data may introduce bias, as participants could underreport or overreport their experiences of workplace violence or burnout. Future research would benefit from longitudinal studies and the inclusion of more diverse, representative samples to further validate and expand upon these findings.

### 5.2 Implications for Nursing and Health Policy

The findings of this study underscore the critical need for comprehensive policies in nursing and healthcare that address workplace violence and its impact on burnout among physicians and nurses in Türkiye. Key implications include the importance of implementing targeted violence prevention programs, enhancing organizational support systems, and fostering a safe and supportive work environment. Additionally, cultural factors and individual experiences must be considered in the development of policies to ensure their effectiveness and relevance. The study highlights the need for hospital management to take proactive measures to inform and protect their staff, which can reduce fear of future violence and improve overall well‐being and job satisfaction.

The data analysis revealed significant differences between the sample groups, nurses and physicians. Overall, nurses faced more intense negative workplace experiences, while physicians had higher concerns about future violence but felt more fulfilled in their roles. Research indicates that nurses generally show positive responses to AI‐enabled tools and improved team efficiency, whereas physicians often experience isolation and exhibit neutral responses to new technologies (Jeong et al. 2024). According to the previous study, nurses benefit most from interventions that enhance team dynamics and workflow efficiency, while physicians respond better to strategies that improve their connectedness within the healthcare team (Clark and Greenawald 2013). Hospital management can employ simulation and instructional training for nurses to foster trust and respect within nursing teams, thereby improving teamwork and communication and ultimately reducing violence. Additionally, nurses can use education and training programs, action plans, and risk assessment tools to help prevent violence. For physicians, modifying workplace design and regulations to mitigate risk factors such as extended wait times and unmet patient expectations can help prevent violence incidents.

## 6 Conclusions

Workplace violence significantly affects healthcare workers in developing countries, contributing to burnout. In Türkiye, despite strong legislation aimed at prevention, healthcare professionals often feel unsupported. Cultural factors, fear, and historical events further exacerbate this issue, negatively affecting their emotional well‐being and sense of personal accomplishment.

This study found that in Türkiye, exposure to violence among healthcare professionals increases burnout symptoms such as depersonalization and emotional exhaustion, yet paradoxically reduces feelings of low personal accomplishment. Moreover, the results indicate that exposure to violence does not significantly impact the fear of future violence among Turkish healthcare professionals. Workplace violence remains prevalent in Türkiye, with factors such as gender, marital status, and previous experiences influencing the fear of future violence. The study also highlights the relevance of CLT in understanding how healthcare professionals perceive and respond to violence, emphasizing the role of cultural norms and prioritization of immediate personal safety concerns.

In that sense, hospital organizations should inform their healthcare personnel about violent events, even if the violence occurs outside their own institutions. Another aspect of the theory emphasizes the importance of understanding how individuals interpret and mentally represent organizational practices, as this can impact their attitudes and behaviors in response to management control systems. Therefore, in the hospital management environment, if nurses perceive management practices against violence as strict and effective, they may experience less fear of future violence. Consequently, hospital management must take necessary precautions and provide ample information to nurses.

## Author Contributions

Study design: IA and ED. Data collection: OC and HSUU. Data analysis: IA. Study supervision: IA, ED, KA, OC, and HSUU. Manuscript writing: IA and KA. Critical revisions for important intellectual content: IA, KA, OC, and ED.

## Conflicts of Interest

The authors declare that there is no conflict of interest.
